# Recruitment to the Nuclear Periphery Can Alter Expression of Genes in Human Cells

**DOI:** 10.1371/journal.pgen.1000039

**Published:** 2008-03-21

**Authors:** Lee E. Finlan, Duncan Sproul, Inga Thomson, Shelagh Boyle, Elizabeth Kerr, Paul Perry, Bauke Ylstra, Jonathan R. Chubb, Wendy A. Bickmore

**Affiliations:** 1 MRC Human Genetics Unit, Institute of Genetics and Molecular Medicine, University of Edinburgh, Edinburgh, United Kingdom; 2 Micro Array Facility, VUMC Cancer Center Amsterdam, Amsterdam, The Netherlands; 3 Division of Cell and Developmental Biology, School of Life Sciences, University of Dundee, Dundee, United Kingdom; The Babraham Institute, United Kingdom

## Abstract

The spatial organisation of the genome in the nucleus has a role in the regulation of gene expression. In vertebrates, chromosomal regions with low gene-density are located close to the nuclear periphery. Correlations have also been made between the transcriptional state of some genes and their location near the nuclear periphery. However, a crucial issue is whether this level of nuclear organisation directly affects gene function, rather than merely reflecting it. To directly investigate whether proximity to the nuclear periphery can influence gene expression in mammalian cells, here we relocate specific human chromosomes to the nuclear periphery by tethering them to a protein of the inner nuclear membrane. We show that this can reversibly suppress the expression of some endogenous human genes located near the tethering sites, and even genes further away. However, the expression of many other genes is not detectably reduced and we show that location at the nuclear periphery is not incompatible with active transcription. The dampening of gene expression around the nuclear periphery is dependent on the activity of histone deacetylases. Our data show that the radial position within the nucleus can influence the expression of some, but not all, genes. This is compatible with the suggestion that re-localisation of genes relative to the peripheral zone of the nucleus could be used by metazoans to modulate the expression of selected genes during development and differentiation.

## Author Summary

Genes are not randomly arranged in the interphase nucleus. In budding yeast, pathways have been characterised that anchor chromatin at the nuclear periphery, and promote the transcriptional repression of loci positioned there. There are also correlations between inactive chromatin and the nuclear periphery in vertebrates. What has been unclear is whether this is cause or effect. Genes might be inactivated by being positioned close to the nuclear periphery or loci inactivated by other mechanisms might just be sequestered there. In this paper, we provide evidence for a causative role of the nuclear periphery in altering gene expression in human cells. We used the interaction between *Eschericia coli* lacO operator sequences, inserted into the human genome, and the lac repressor protein, fused to a protein of the inner nuclear membrane, to reposition two different regions of the human genome to the nuclear periphery. The expression of some, but not all, genes on the relocated chromosomes was suppressed. We also show directly that location adjacent to the nuclear periphery is not incompatible with active transcription. Therefore it is possible that positioning of genes relative to the nuclear periphery could be used as a mechanism to modulate gene expression in vertebrates.

## Introduction

The role of chromatin structure in regulating gene expression is undisputed and increasingly well understood. What is less clear is whether the spatial organisation of the genome in the nucleus also acts to modulate gene expression. In mammals, regions with low gene-density, and that are late-replicating, concentrate toward the periphery of the nucleus [1]–[7]. Inactive transgenes[8] and some endogenous inactive genes[9]–[11] also locate close to the nuclear periphery and their movement away from there correlates with their transcriptional activation [12]. However, the expression of many other genes in mammalian cells appears unaffected by their proximity to the nuclear periphery [13]–[15]. Therefore, a crucial issue is whether, and to what extent, this level of nuclear organisation directly affects gene function rather than merely reflecting it.

In the budding yeast *Saccharomyces cerevisiae (S. cer.)*, silent chromatin is anchored at the nuclear periphery and this, in turn, promotes transcriptional repression [16],[17]. Ku, Sir4 and Esc proteins mediate the anchoring of silent loci at the nuclear periphery [18],[19]. However, in mammals Ku is predominantly a nucleolar protein[20],[21] involved in double-strand break repair, and there are no direct mammalian Sir4 and Esc homologues at the nuclear periphery. Yeast also does not appear to have the extensive array of integral membrane proteins and lamins that are present at the periphery of the mammalian nucleus. Therefore, it is unclear whether proximity to the nuclear periphery plays any role in the regulation of mammalian gene expression.

Consistent with the idea that the mammalian nuclear periphery is involved in the regulation of gene expression are the many reports that lamins and several of the inner nuclear membrane (INM) proteins interact with, or sequester, proteins that regulate transcription. Some genes on chromosomes that move away from the nuclear periphery in murine cells mutant for Lamin B1, or Lamin B1 processing, are transcriptionally upregulated [22]. In addition, the LEM domain-containing INM proteins Lap2β, emerin and MAN1, interact with a range of transcriptional regulators. MAN1 interacts with R-Smads, germ-cell-less (GCL), barrier-to-autointegration factor (BAF) and BCL-2 associated transcription factor (BTF) [23]. Emerin interacts with BAF, BTF, GCL, histone deacetylases (HDACs), the nuclear corepressor (NCoR) complex and with β-catenin [24],[25] and Lap2β binds lamin B, chromatin, GCL and HDACs [26]–[28].

To investigate whether proximity to the nuclear periphery can directly facilitate transcriptional suppression in mammalian cells, here we relocate two different human chromosomes, tagged with arrays of the *Eschericia coli* (*E.coli*) lac operator (lacO) [6], to the nuclear periphery via interaction with lac repressor (lacI) that is fused to the integral INM protein Lap2β. We show that this can reduce the expression of some endogenous human genes located near the lacO sites. Some genes very far from lacO also appear to be down-regulated by relocalisation of the tagged chromosome toward the nuclear periphery. Dampening of gene expression close to the nuclear periphery is reversed if either the activity of histone deacetylases (HDACs), or the tethering mechanism, is blocked. Importantly, the expression of many genes near the tethering sites is not detectably reduced by their altered nuclear position and indeed we directly show that location at the nuclear periphery is still compatible with active transcription from a reporter gene.

These data suggest that, during development and differentiation, re-localisation relative to the nuclear periphery could be used to modulate the expression of certain genes without necessarily altering expression of their neighbours.

## Results

### Experimental Tethering of Different Human Chromosomes to the Nuclear Periphery

To directly determine whether proximity to the nuclear periphery can mediate gene suppression in mammalian cells we tethered two different human chromosomes to the INM in human HT1080 cells. Previously, we had randomly integrated a 128-mer array of the *E.coli)* lacO sequence into the genome of HT1080 cells and characterised single site insertions. Interphase fluorescence in situ hybridisation (FISH) had also shown that these integrations do not detectably perturb the radial nuclear disposition of human chromosomes [6]. Integrations into 4q28 and 11q13 were identified in cell lines; B49.5 and J21.C3, respectively, by FISH on metaphase chromosomes (data not shown).

**Figure 1:**
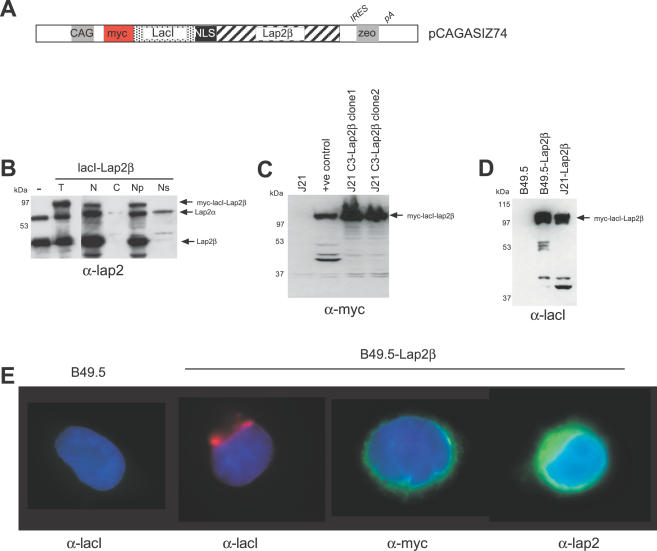
Establishing the lacO/lacI Tethering System. (A) Map of pCAGASIZ74 encoding myc-lacI -lap2β under the control of the CAG (CMV-chicken actin) promoter. (B) Western blot of fractionated extracts from cells transfected with pCAGASIZ74 and detected with antibody that recognises all isoforms of lap2, - untransfected cells; T- total cell extract; N –nuclear extract; C – cytoplasmic extract; Np – insoluble nuclear extract; Ns – soluble nuclear extract. (C) Western blot with antibody detecting the myc tag on cell lines with lacO sites in chromosome 11 (J1-C3) and stably expressing the myc-tagged lacI-lap2β; J21 = lacO integrated cells without lacI-lap2β, +ve control = transiently transfected cells. (D) Western blot with antibody detecting lacI in the lacO integrated parental B49.5 cells, and in B49.5 and J21 cells stably expressing lacI-lap2β. (E) Immunofluorescence on parental B49.5 lacO integrated cells and B49.5 cells stably expressing lacI-lap2β with antibodies that recognise lacI, myc or lap2. Nuclei are counterstained with DAPI.

To tether these lacO integration sites to the nuclear periphery, we made fusion constructs between *E. coli* lacI, which binds with high affinity to lacO sequences, and the mammalian integral INM protein Lap2β. Fusion of lacI was to the N-terminus of this type II membrane protein as this is the end of the protein that faces the nucleoplasm [29], and a myc-tag was placed N-terminal of lacI to aid immunodetection (Figure 1A). Subcellular targeting of the tethering protein was first analysed by Western blot of fractionated cell extracts from transfected cells. The partitioning of the tethering protein into the insoluble fraction of the nucleus mirrored that of endogenous Lap2β and is consistent with its insertion into the nuclear membrane. This contrasts with the larger (α) isoform of endogenous Lap2 (Lap2α) that lacks a transmembrane domain, is nucleoplasmic[29] and that was present in both the insoluble and the soluble fractions of the nucleus (Figure 1B). Expression of the tethering construct in stable selected cell lines was assessed by Western blot, using antibodies that detect myc (Figure 1C) and lacI (Figure 1D). We estimated that this fusion protein is present at ∼50% of the levels of endogenous Lap2β (data not shown). Immunofluorescence, with antibodies detecting lacI, myc and Lap2, visually confirmed that lacI-lap2β protein was located at the nuclear periphery, with some protein also in the endoplasmic reticulum (Figure 1E).

**Figure 2:**
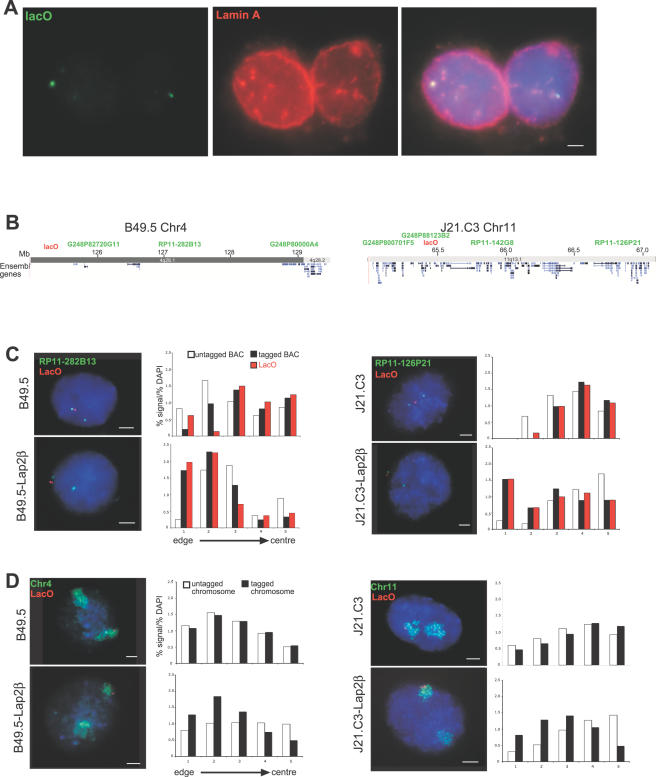
Subnuclear Localisation of lacO-Tagged Chromosomes. (A) 3D immuno-FISH on B49.5 cells expressing lacI fused to an INM protein, using a DNA probe for lacO and antibody detecting Lamin A. The single image plane shows lacO signals co-localised with foci of lamin A that are at invaginations of the nuclear envelope. Scale bar = 2 µm. (B) Approximate sites of lacO integration (red), determined from interphase FISH with genomic clones (green), on chromosomes 4 and 11 in cell lines B49.5 and J21.C3, respectively. Map position and location of genes is taken from the March 2006 (NCBI Build 36.1) Assembly of the human genome at UCSC (http://genome.ucsc.edu/cgi-bin/hgGateway). Details of clone positions are given in Table S1. (C) Interphase FISH with probes for lacO (red) and neighbouring BAC clones (green) in (top row) lacO tagged cell lines B49.5 and J21.C3 and (bottom row) these same cells lines now expressing lacI-lap2β. Scale bars = 2 µm. To the immediate right of each FISH image the histograms quantify the mean proportion of probe hybridisation signal, normalised to the proportion of DAPI stain (y axis), across the 5 concentric shells eroded from the periphery (shell 1) through to the centre (shell 5) of the nucleus (x axis), for the proximal BAC on the untagged chromosome (open bars) and on the tagged chromosome (black bars) and the lacO sites (red bars) in each of the parental and lacI-lap2β expressing cell lines. n = 35–50 for each cell line. (D) as in (C) but using probes for lacO (red) and chromosome paints (green).

To determine whether the lacI-Lap2β fusion protein was able to tether lacO integration sites to the nuclear periphery, 3D immuno-FISH was performed on B49.5 cells expressing tethered lacI. Analysis of these cells revealed that 89% of lacO signals co-localised with signal for lamin A (n = 62). In many cases, this colocalisation did not appear to be exactly at the edge of the nucleus (Figure 2A). However, closer examination through the z-axis of these cells revealed that these foci of lamin A represent invaginations of the nuclear envelope. Such invaginations are commonly seen in cell lines derived from tumours[30] (including the HT1080 cells used here – our unpublished observations).

To examine whether the chromosomal region around the site of lacO integration was also relocated toward the nuclear periphery, we used genomic clones (Table S1) in interphase FISH to determine those probes located close to the lacO signal (Figure 2B). FISH, with probes for lacO and a proximal genomic clone, was then performed on nuclei from the original lacO tagged cell lines, and from their counterparts now expressing the lacI-Lap2β tethering protein. This enabled the nuclear position of the lacO-tagged chromosome to be directly compared with the homologous untagged (wild-type) chromosome in the same nucleus (Figure 2C). The radial position of these probes was established from the distribution of hybridisation signals, relative to that of total DNA, amongst five shells of equal area eroded from the edge (shell 1) through to the centre (shell 5) of the nucleus. Even though this is a 2D analysis it can be used to infer 3D organisation [1],[2]. In both starting cell lines, the radial distribution of the chromosome carrying the lacO integration was similar to that of the equivalent untagged chromosomes in the same nucleus (p>0.13 by chi-square test) (Figure 2C). The lacO integration site on chromosomes 4 (B49.5 cells) was closer to the nuclear periphery than the site on chromosome 11 in J21.C3 cells. This reflects the known radial organisation of human chromosomes with differing gene densities; the density of genes on human chromosome 11 is more than twice that on chromosome 4 (10.8 genes/Mb compared with 4.5) [2]. In the equivalent cell lines now expressing lacI-Lap2β, both the lacO and proximal genomic probe signals detecting the lacO-tagged chromosomes 4 or 11 appear to be displaced toward the nuclear periphery compared to their wild-type counterparts in the same cells and indeed the untagged and tagged loci now have significantly different nuclear distributions from each other (p<10^−6^) (Figure 2C).

We then ascertained whether it is just the immediate vicinity of the lacO integration site that is recruited to the nuclear periphery, and so pulled out of the host chromosome territory, or whether there is a nuclear redistribution of the entire lacO tagged chromosome in the presence of lacI-Lap2β (Figure 2D). Using interphase FISH with paints for human chromosomes 4 and 11 we determined that, as for the analysis with BACs in Figure 2C, there is no significant difference in the nuclear distributions of the tagged and untagged human chromosomes in the parental cell lines (p>0.5). But in the presence of lacI-Lap2β there is a significant relocation of the whole lacO-tagged chromosome towards the nuclear periphery, compared with the position of the homologous untagged chromosome in the same cell nucleus (p<10^−5^ for chromosome 4 in B49.5 cells and p = 0 for chromosome 11 in J21.C3 cells). Therefore we conclude that the lacI-Lap2β protein inserted in the INM can bind to lacO arrays integrated into human chromosomes and so retain these chromosomes toward the nuclear periphery. Given the limited range of chromatin motion in interphase [6], and the fact that INM-derived vesicles first accumulate on chromosomes at anaphase, we considered it likely that chromosome recruitment to the nuclear periphery is established as cells exit mitosis [31]. Indeed, a recent study using an inducible tethering to a nuclear lamin, has shown that relocalisation occurs as cells exit telophase and enter G1 [32].

### Gene Expression Is Altered on Human Chromosomes Tethered to the Nuclear Periphery

**Figure 3:**
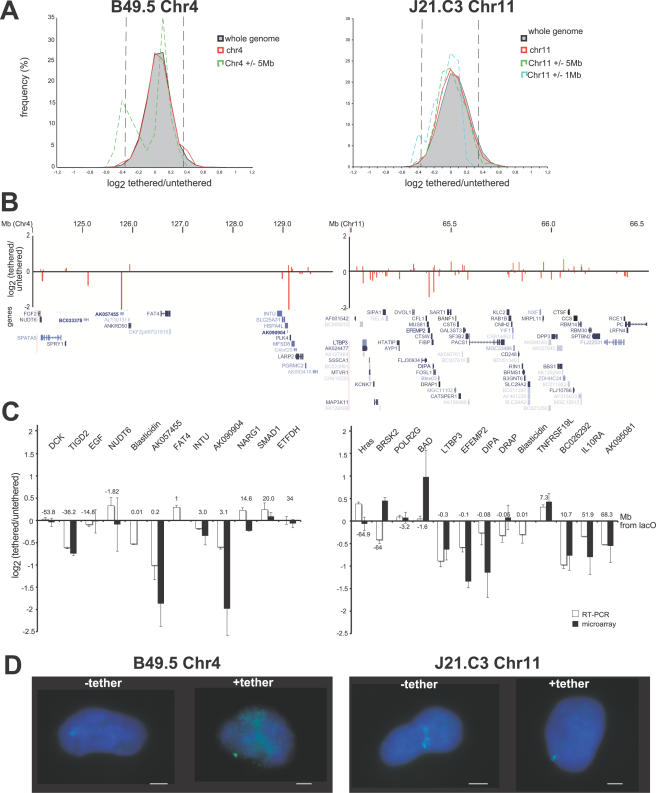
Gene Expression Changes in lacO-Tagged Cell Lines. (A) Frequency histograms of log_2_ tethered/untethered (i.e +/−lacI-lap2β) cDNA ratios for a moving average of 5 genes in cell lines B49.5 (left) and J21.C3 (right). Data for the whole genome (shaded in grey) are compared to the data from the whole of the respective tethered chromosome (red line) or for windows of +/−5 and 1 Mb (green and blue dashed lines respectively) centered around the site of lacO integration in each cell line. Dashed lines indicate the 99% confidence limits set from self-self hybridisations. (B) Mean log2 tethered/untethered (i.e +/−lacI-lap2β) cDNA ratios for genes in the 6 Mb near the lacO site on chromosome 4 in B49.5 cells (left) or the 1.5 Mb region close to lacO on chromosome 11 in J21.C3 cells (right). Mapping data and gene positions are relative to the May 2004 assembly of the human genome in the UCSC browser. (C) qRT-PCR analysis of endogenous genes on chromosome 4 (left) or chromosome11 (right) as well as the blasticidin gene that is in cis with the lacO sites. Graphs show the mean (+/−s.e.m) log2 tethered/untethered (i.e +/−lacI-lap2β) ratios from independent analyses, normalised to β-actin (white bars). Black bars indicate the log2 ratios obtained from the microarray analysis. Values along the x axis indicate estimated distance from lacO integration site in Mb (+values are telomeric of the integration site, −ve values are centromeric). (D) RNA FISH for blasticidin transcript (green) on B49.5 and J21.C3 untethered cells and the corresponding cell lines expressing lacI-lap2β (tethered). Scale bars = 2 µm.

A microarray hybridisation approach was first used to analyse any changes in gene expression that occur as a consequence of tethering lacO sites on chromosomes 4 and 11 at the nuclear periphery. RNA prepared from the untethered starting lacO integration cell lines was compared to that from the equivalent cell line now expressing the lacI-Lap2β tether. Log_2_+/−lacI-lap2β ratios from four replicate hybridisations (including both biological and technical replicates) were analysed as a running mean of 5 genes across the whole genome. Self-self hybridisations were used to establish that the 99% confidence intervals (CI) on these data are log2+/−0.35 [33]. There was no evidence for chromosome-wide suppression of gene expression on the relocated chromosome 4 in B49.5 lacI-lap2β tethered cell lines (Figure 3A). However, tethering to the nuclear periphery is associated with a domain of down-regulation (>99% CI) of gene expression in the vicinity (+/−<5 Mb) of the lacO array at 4q28.

Closer examination of the data from the region near the lacO integration site indicates significant down-regulation at three genes; *BC033378* (p = 0.015), *AK057455* (p<10^−4^) and *AK090904* (p<10^−4^) that are spread over a 4 Mb gene-poor region (Figure 3B). However, the expression of other genes in the region appears unaffected by tethering. Real time RT-PCR (qRT-PCR), normalising expression levels relative to those for β-actin, was used to confirm the changes in expression of these genes, and of genes selected from other regions of the tethered chromosome (Figure 3C). The level of repression of *AK057455* revealed by this analysis would be consistent with the complete silencing of this gene on the tethered chromosome (log2 = −1), since there is a wild-type (untagged) homologous chromosome in these cell lines (Figure 2). Expression of *AK090904* appears reduced, but not completely silenced.

In addition qRT-PCR analysis allowed us to analyse expression from the blasticidin selection cassette that was inserted in cis during the original selection of the lacO arrays [6] and that was not represented on the microarrays. We observed a 20–30% reduction in expression of the blasticidin reporter gene in the tethered cell lines, and this was irrespective of whether blasticidin selection was maintained on the cells or not. It seemed unlikely that remaining blasticidin expression could be from the estimated 11% (Figure 2A) of alleles that are not co-localised with components of the nuclear periphery. It is more likely that there is still some expression of the blasticidin reporter gene on the tethered chromosomes. Indeed, RNA FISH revealed sites of blasticidin nascent transcription in 61% of tethered B49.5 cells and 60% of J21.C3 cells (n = 1200 cells in each case). This compared with detection rates by RNA FISH of 75% and 83% in untethered B49.5 and J21.C3 cells, respectively. For the integration on chromosome 11 in J21.C3 cells, we assessed that 48% of the RNA FISH signals were close to the nuclear periphery in the tethered cell line, compared with only 19% of signals in the untethered cell line (n = 300) (Figure 3D). We conclude that although tethering at the nuclear periphery reduces expression of some endogenous human genes, it is still compatible with transcription, especially from a gene with a strong promoter (the blasticidin gene is driven by an SV40 viral promoter) [6].

The altered gene expression is due to the presence of the lacI-lap2β tethering protein, and not simply due to lac repressor binding, since there is no transcriptional suppression in cells expressing a free nucleoplasmic lacI (Figure S1A) [6]. Suppression of endogenous genes on chromosome 4 in cells expressing lacI-lap2β is dependent on the lacO arrays being in cis in the B49.5 line, since it is not seen in J21.C3 cells in which the lacO arrays are on chromosome 11 (Figure S1B). Blasticidin expression is still reduced in this analysis because this reporter is on chromosome 11 in J21.C3 cells. These data indicate that suppression is not a non-specific effect of the expression of lacI-lap2β. (Figure S1B).

In addition, microarray analysis provided some evidence (p<0.01) for reduced gene expression at a few other sites (running average of 5 genes) on chromosome 4 (at positions ∼4, 15, 42, 72, 90.5, 107.5, 185 Mb) that are distant from the lacO integration at position ∼129 Mb. Real-time RT-PCR confirmed the down-regulation of a gene (*TIGD2*) from one of these sites (the cluster at position 90.5 Mb in 4q22) (Figure 3C).

In J21.C3 cells, lacO arrays are integrated into a very gene-rich area of chromosome 11q13. Tethering of this chromosome to the nuclear periphery is also associated with a domain of significant gene suppression (>99% CI for sliding windows of 5 genes) in the 1 Mb around the lacO integration site (Figure 3A). Closer examination reveals significantly reduced expression of multiple genes (*LTBP3*, p = 0.03; *EFEMBP2*, p<10^−4^; *DIPA*, p<10^−4^) in the region close to the lacO integration site (Figure 3B) and this was also confirmed by qRT-PCR (Figure 3C). Decreased expression from the blasticidin reporter on chromosome 11 in this cell line was also detected by qRT-PCR, consistent with RNA FISH analysis. However, as for the analysis of chromosome 4 genes in B49.5 cells, there are also genes in the vicinity of the lacO arrays on 11q13 whose expression is not detectably reduced by tethering at the nuclear periphery in J21.C3 cells (Figure 3B). There also appears to be significant (p<0.01) down-regulation of several clusters of genes (at positions 4.5, 22.5, 45, 109 and 117.5 Mb on chromosome 11), located far away from the lacO site at position ∼65.5 Mb. Real-time RT-PCR confirmed the down-regulation of a gene (*IL10RA*) from one of these sites (the 117.5 Mb cluster) (Figure 3C).

By qRT-PCR, there was no change in expression of the tested chromosome 11 genes in J21.C3 cells expressing a free nucleoplasmic lacI (Figure S1A), and the extensive suppression of groups of genes on chromosome 11 is not seen in B49.5 cells (where lacO is on chromosome 4) (Figure S1B). We conclude that tethering to the INM induces a dampening of transcription of many genes in the vicinity of the tethering site on chromosomes 4 or 11, and also of some genes located elsewhere on these chromosomes.

### A Role of Histone Deacetylation in Transcriptional Suppression at the Nuclear Periphery

**Figure 4:**
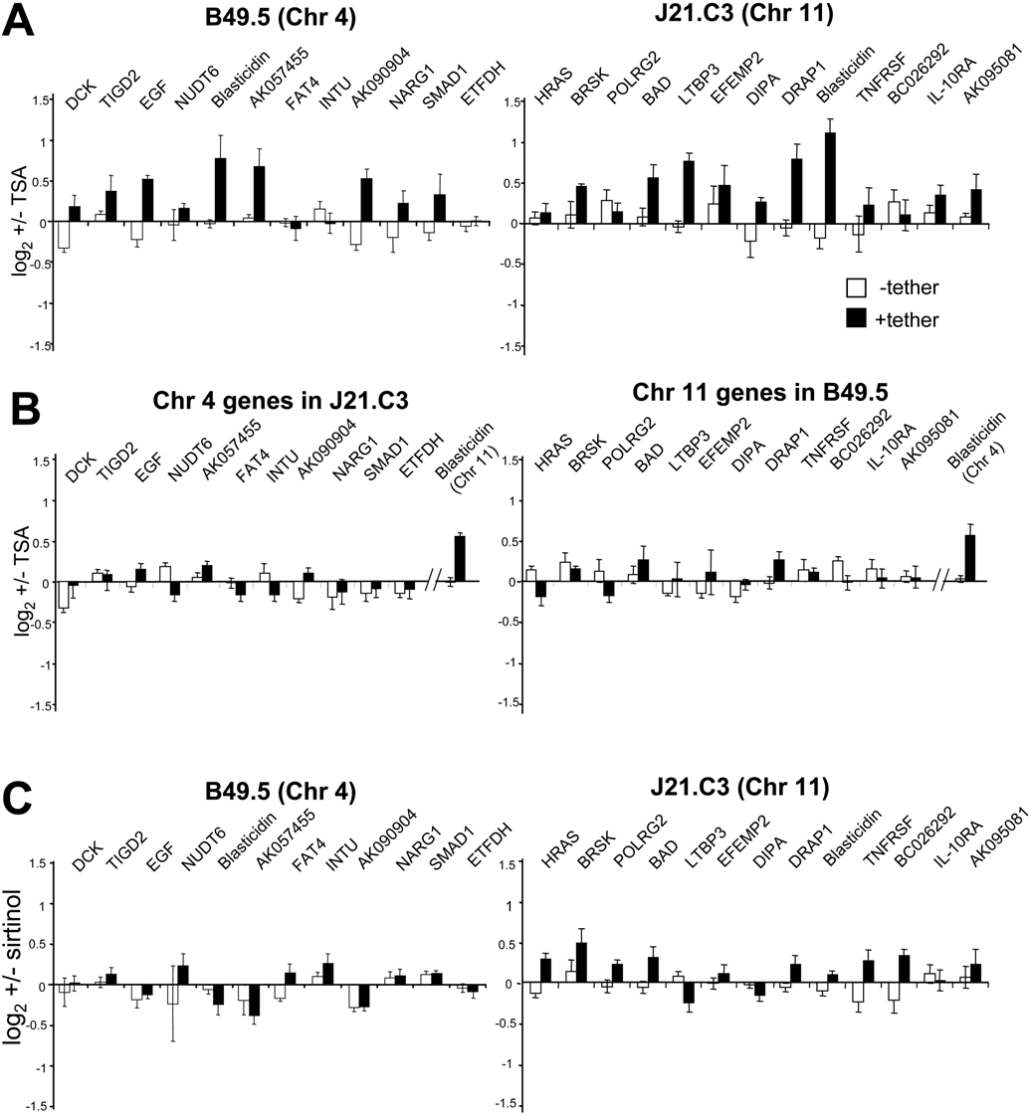
Influence of Histone Deacetylase Inhibitors. (A) qRT-PCR analysis, of endogenous genes on chromosome 4 in B49.5 cells (left) or genes on chromosome11 in J21.C3 cells (right), as well as the blasticidin gene that is in cis with the lacO sites, in mock-treated (DMSO) or TSA treated cells. Graphs show the mean (+/−s.e.m) log2 +/− TSA ratios from independent analyses, normalised to β-actin. Black bars; data from cells expressing the lacI-lap2β tether, white bars; parental cells with no tethering protein. (B) qRT-PCR analysis of endogenous genes on chromosome 4 in J21.C3 cells (left) or genes on chromosome11 in B49.5 cells (right) in mock-treated (DMSO) or TSA treated cells. (C) As in (A) but after treatment with sirtinol.

What might be the mechanism of transcriptional suppression caused by retention of chromosomes to the periphery of the human nucleus? In budding yeast, it is suggested that loci at the nuclear periphery are exposed to high concentrations of Sir proteins, and that this is what promotes repression [18],[19]. Sir2 is an NAD+ dependent class III HDAC. Although there is no Sir2 orthologue reported at the nuclear periphery of mammalian cells, several INM proteins, including emerin [24] and Lap2β [27], interact with the class II HDAC3. There is a zone of histone hypoacetylation around the periphery of the mammalian nucleus [7], consistent with a concentration of HDAC activity there and treatment of cells with trichostatin A (TSA) which inhibits class I and II, but not class III, HDACs elevates levels of histone acetylation, especially around the nuclear periphery [34]. Therefore we analysed what happened to the expression of the down-regulated genes in tethered cell lines after treatment with TSA or with the class III HDAC inhibitor sirtinol. Western blotting confirmed the increased levels of global histone acetylation in treated cells (Figure S2A), and DNA FISH showed that the lacO-tagged chromosomes remain at the nuclear periphery in treated cells (Figure S2B). By qRT-PCR, expression of the blasticidin reporter, and of endogenous genes both near to, and distant from, the lacO sites, was increased in TSA treated cells compared to untreated controls. This was dependent on the presence of the tethering protein (+lacI-lap2β) as it was not seen in the untethered cell lines (Figure 4A). Chromosome 4 genes were not affected in TSA treated J21.C3 cells, and chromosome 11 genes were unaffected in TSA treated B49.5 cells, indicating that the TSA affects only genes linked in cis to the tethered lacO (Figure 4B). An apparent decrease in expression of genes in the untethered cell lines may reflect a small effect of TSA on the relative levels of β-actin expression, which was used to normalise expression levels in qRT-PCR. Sirtinol treatment had no significant effect on genes close to the lacO arrays, including the blasticidin reporter, in either cell line (Figure 4C). We conclude that the reduced expression of genes on the tethered chromosomes is likely due, at least in part, to the activity of class I/II HDACs located in a zone at the periphery of the nucleus.

### Disrupting the Tethering Mechanism Reverses Gene Suppression

**Figure 5:**
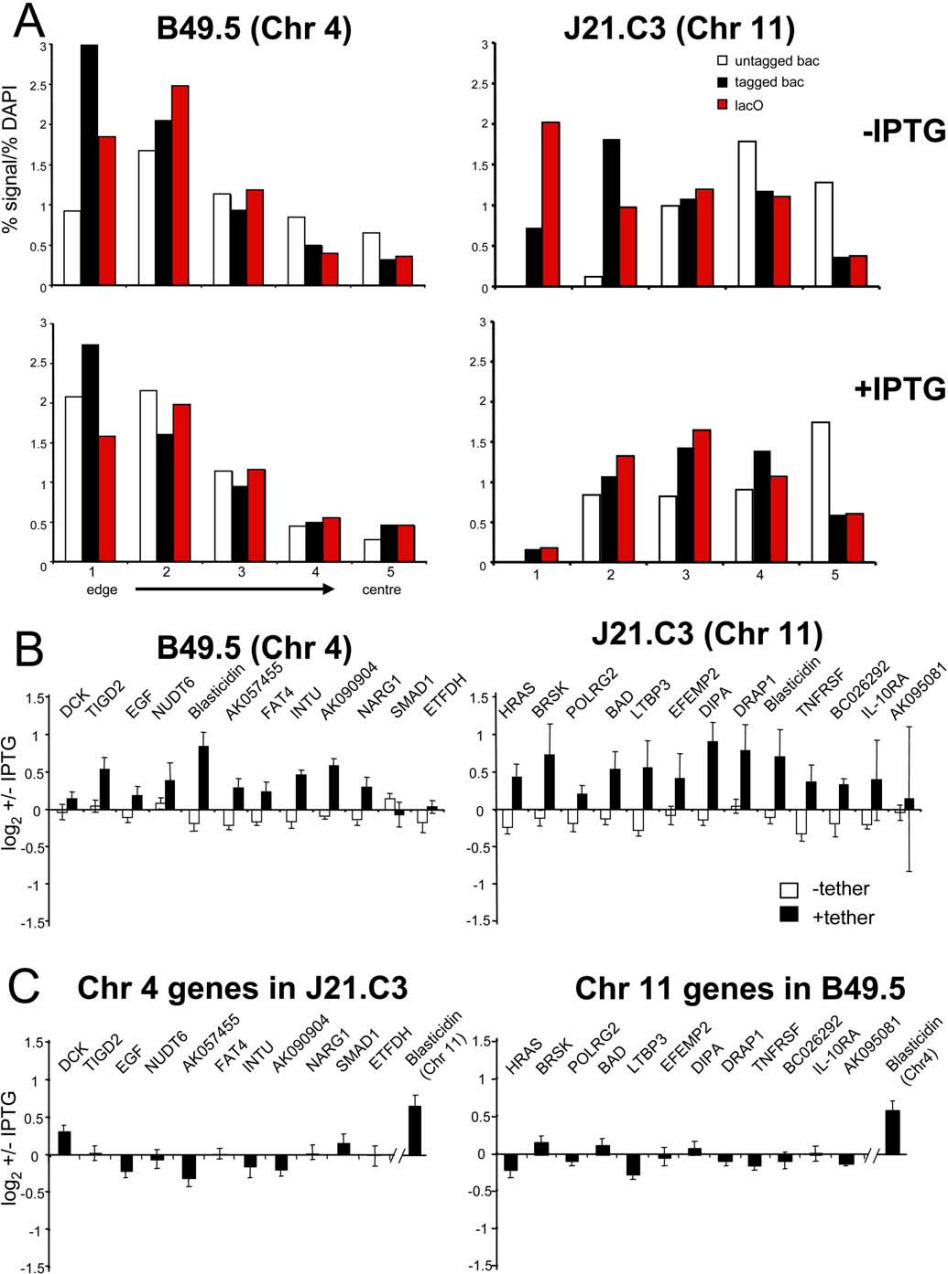
Reversibility of lacO-lacI Tethering and Gene Suppression. (A) Histograms showing the mean proportion (%) of probe hybridisation signal, normalised to the proportion of DAPI stain (y axis), across the 5 concentric shells eroded from the periphery (shell 1) through to the centre (shell 5) of the nucleus (x axis), for a proximal BAC on the untagged chromosome (open bars) and on the tagged chromosome (black bars) and the lacO sites (red bars) in the lacI-lap2β expressing cell lines grown for 48 hours with (+) and without (−) IPTG. n = 50 for each cell line. (B) qRT-PCR analysis of expression from endogenous genes on chromosome 4 in B49.5 cells (left) or on chromosome11 in J21.C3 cells (right), as well as the blasticidin gene, in cells grown with (+) and without (−) IPTG. Graphs show the mean (+/−s.e.m) log2 +/− IPTG ratios from independent analyses normalised to β-actin. Black bars; data from cells expressing the lacI-lap2β tether, white bars; parental cells with no tethering protein. (C) qRT-PCR analysis of genes on chromosome 4 in J21.C3 cells (left) or genes on chromosome11 in B49.5 cells (right) expressing the lacI-lap2β tethering protein and treated with IPTG.

To determine whether, once established, gene suppression and peripheral localisation can be maintained in the absence of lacO/lacI mediated tethering, we treated both tethered and untethered cell lines for 48 hours with 4 mM isopropyl β-D-1 thiogalactopyranoside (IPTG). This abrogates the interaction between lacO and lacI, and erosion analysis (Figure 5A) showed that this treatment altered the relative nuclear positions of the lacO-tagged chromosomes. In the J21.C3 cell line the tagged chromosome 11 was relocated away from the nuclear periphery in IPTG treated cells, compared to its position in untreated cells, although it still remained significantly more peripherally located than the untagged chromosome 11 (Figure 5A). In the case of the tagged chromosome 4 in B49.5 cells, a movement of the tagged chromosome away from the nuclear periphery (as defined by erosion analysis) was less apparent after IPTG treatment, but there was no longer any significant difference in the radial nuclear distribution of the loci assayed on the tagged and untagged chromosomes 4 in the treated cells (p = 0.1), compared with the significant difference in untreated cells (p<10^−5^). Therefore we conclude that the differential nuclear positions of lacO-tagged and untagged chromosomes requires the persistence of the lacO/lacI interaction, but that the return of the lacO-tagged chromosomes to their normal nuclear position (i.e. the position in cells that never expressed lacI-lap2b, Figure 2C) is slow, and might require more than once cell cycle.

Surprisingly, we also noted that IPTG treatment resulted in an apparent nuclear redistribution of the untagged chromosome 4 locus of B49.5 cells towards the outer erosion shell 1 compared to untreated cells. This effect can also be seen when comparing the position of this locus in the parental B49.5 cell line (no lacI-lap2b fusion) to that in the equivalent tethered cell line (Figure 2C). This could be due to the lacO-lacI interaction inducing an increased frequency or extent of invaginations of the nuclear membrane (which are not taken into account in simple 2D erosion). Alternatively, it may reflect a real displacement, by the tagged chromosome 4, of the untagged chromosome away from the nuclear periphery. There is limited space at the nuclear periphery, so strong artificial tethering of a large chromosome there may necessitate other parts of the genome being moved out of the way and chromosome 4 is normally found quite close to the nuclear periphery.

qRT-PCR analysis revealed that IPTG increased the expression of genes whose expression had been reduced as a consequence of lacI-lap2β-mediated tethering to the nuclear periphery, in both the B49.5 and J21.C3 cell lines (Figure 5B). IPTG had no affect on these genes in the parental cell lines that do not express lacI-lap2β. Analysis of chromosome 4 genes in J21.C3 treated cells, and chromosome 11 genes in B49.5 treated cells, showed that IPTG only affects genes in cis with the lacO arrays (Figure 5C). Therefore we conclude that gene suppression at the nuclear periphery is not heritable, but rather depends on active tethering of the chromosome to the nuclear periphery.

## Discussion

Here we have demonstrated that the expression of some human genes is dampened by recruitment of their host chromosomes to the nuclear periphery, mediated via binding of integrated lacO arrays to a fusion protein between lacI and the INM protein Lap2β. The fact that a window of significant transcriptional suppression is seen over a large genomic region in the vicinity of the lacO arrays (Figure 3B), together with the non-contiguous nature of the suppressed genes, makes it unlikely that the mechanism is some kind of molecular crowding/exclusion at the nuclear periphery in the immediate vicinity of the lacI/lap2β fusion protein.

We have detected down-regulation of genes on two different tethered human chromosomes (4 and 11). The most potent transcriptional dampening is focussed at genes close to the lacO tethering site, but there was also evidence for significant down-regulation of expression at several other sites along the tethered chromosomes. We suggest that this transcriptional suppression is mediated, at least in part, by the action of class I/II HDACs in a zone of histone hypoacetylation found around the nuclear periphery of mammalian cells [7],[34]. This is supported by the observation that TSA treatment leads to an increase in histone acetylation levels at the nuclear periphery [34] and that treatment with this HDAC inhibitor relieves the transcriptional suppression of genes on chromosomes tethered to the nuclear periphery (Figure 4A). Indeed, the INM proteins emerin and Lap2β have been shown to interact with HDAC3 [24],[25],[27].

The magnitude of change in gene expression on tethered chromosomes (Figure 3C) is generally consistent with reduced transcriptional activity rather than with a complete gene silencing (which would be represented by a log2 = −1). Similarly, the steady state levels of mRNA from the blasticidin reporter gene, where there is no allele on the untagged chromosomes, are reduced by only ∼20–30% (Figure 3C) and by RNA FISH we show that blasticidin alleles in the tethered cell lines are still able to be transcribed, albeit at reduced efficiency (Figure 3D). This is consistent with a recent study in which a reporter cassette was targeted via lacO-lacI interaction to a component of the nuclear lamina, a structure which underlies the nuclear membrane [32]. In that case, nascent transcription of the reporter was also detected at the nuclear periphery, and the efficiency of this also appeared reduced in the targeted cell lines compared to controls. In our analysis here, both microarray analysis and qRT-PCR were unable to detect any reduction in the expression for many other endogenous human genes close to the lacO tethering sites on chromosome 4 and 11. This is entirely consistent with the fact that many poised or active loci are found located at the periphery of the mammalian nucleus [13]–[15]. Therefore, we conclude that the environment close to the nuclear periphery is able to impact on the expression of some genes, but not on others, and to variable extents. This may reflect differences in promoter strength amongst genes, for example blasticidin expression is being driven by a strong viral (SV40) promoter, or it may reflect interplay between the nuclear environment and pre-existing chromatin marks (e.g. histone modifications) at the different gene loci.

The data that we have presented here are consistent with correlations that have been made between gene expression and gene localisation in the mammalian nucleus either between cell types [9],[10], or during differentiation [11]. Therefore we suggest that the re-localisation of genomic regions relative to the peripheral zone of mammalian nuclei could be used, in concert with other mechanisms of chromatin-mediated gene regulation, to modulate the expression of selected genes. This is congruent with the observation that during neural differentiation of mouse embryonic stem cells, a large region of chromosome 10 moves from the nuclear periphery to a more internal nuclear position [11]. This is accompanied by strong transcriptional up-regulation and a switch to earlier replication time, of a gene in this region (*Mash1*) that encodes a key neural transcription factor. Whereas some other genes in this region are also up-regulated along with Mash1 during neural differentiation, expression of other genes in this region is unaffected by their altered nuclear positioning.

More recently, location close to nuclear pores complex components has been correlated with an up-regulation of gene expression in yeast and Drosophila [35]. Indeed, in our data we have noted groups of genes whose expression appears to increase upon tethering toward the nuclear periphery, though at present we do not know whether this is a direct or indirect effect (data not shown). It will be interesting to investigate this further, and to use this experimental system to now investigate how human genes behave when they are tethered to different components of the nuclear periphery, including the nuclear pore complex. It seems likely that the nuclear periphery provides a complex environment for the modulation of gene expression in mammals in normal development and differentiation, and in disease.

## Materials and Methods

### Cell Culture, Treatments, and Transfection

Transfections into HT1080 cells were performed using Lipofectamine 2000 (Invitrogen) as per the manufacturer's instructions. LacO integrations were selected for using 5 µg/ml blasticidinS as previously described [6]. Tethering constructs were linearised with PvuI prior to transfection into the lacO integrant cell lines and colonies were selected with 220 µg/ml zeocin.

To inhibit HDACS, ∼4×10^6^ cells were plated into 10 cm^3^ petri dishes with 10 ml of supplemented DMEM. After 24 hours, cells were treated with 0.5% DMSO in DMEM (mock), 10 µM Sirtinol (Sigma: S7942) or 1 µM TSA (Sigma: T8552) dissolved in 0.5% DMSO/DMEM and further incubated for 8 hours (cells were 60–70% confluent at harvest point) [34],[36].

To abrogate lacO-lacI interactions, cells plated into 10 cm^3^ petri dishes at 50% confluency were cultured in medium containing 4 mM IPTG (Fluka chemicals, UK) for 48 hours, before harvest.

### Plasmid Constructs

The initial vector backbone for expression of the lacI anchoring proteins was pDsRed1-N1 (Clontech) from which the DsRed had been removed by digestion with BamHI and NotI, followed by filling-in and religation (JRC57). A fusion of the myc-tag, lacI-NLS and rat Lap2β was then inserted into the HindIII-KpnI site of JRC57, to make pmyc-lacI-NLS-lap2β (JRC74). An MluI/SmaI fragment from JRC74 that carries the fusion protein cassette was then subcloned into pCAGASIZXN (gift from A. Smith) to make pCAGSIZ74 (Figure 1A). This allows for stable transgene expression from the CAG (CMV-chicken actin) promoter since the selection cassette containing the zeo (*Streptoalloteichus* bleomycin resistance) gene is encoded in a bicistronic mRNA downstream of the tethering construct and is translated from an internal IRES [37]. The free nucleoplasmic lacI vector was created by modification of p3′-SS-GFP-LacI-NLS [6]. Deletion of the GFP tag (750b.p) was achieved by double restriction digest using XbaI and Bsrg1 to generate cohesive ends. Gel purified plasmid, minus GFP, was then religated to generate an in frame LacI-NLS.

### Western Blotting and Nuclear Fractionation

Western blot analysis was carried out using standard protocols. Lap2β was detected with a mouse monoclonal antibody [6E10] that detects all Lap2 isoforms (Abcam, ab11823, 1∶500 dilution), myc was detected with monoclonal 9E10 (Upstate, #05-419, 1∶500) and lacI with monoclonal 9A5(Upstate, #05-503, 1∶500). To analyse histone acetylation levels, rabbit polyclonal anti-H4K5ac (Upstate #H30417, 1∶4000 dilution) and anti-panH4 (Abcam, #7311, 1∶125 dilution) antibodies were used. Goat antibodies to mouse (#A9917) and to rabbit (#A0545) conjugated with horseradish peroxidase were used as secondary antibodies at 1∶1000 dilutions (Sigma). Sub-cellular fractionation was by Dounce homogenization of cells in 10 mM HEPES (pH 7.9); 1.5 mM MgCl_2_; 10 mM KCl; 0.2 mM PMSF, 0.5 mM DTT; 1× protease inhibitor cocktail (Invitrogen). Nuclei were pelleted at 3300 g for 15 mins and the supernatant was taken as the cytoplasmic fraction. The nuclei were then resuspended in 1 packed nuclear volume (pnv) of low salt buffer (20 mM HEPES [pH7.9]; 25% glycerol; 1.5 mM MgCl_2_; 0.02 mM KCl; 0.2 mM EDTA; 0.2 mM PMSF, 0.5 mM DTT; 1× protease inhibitor cocktail). 1pnv of low salt buffer containing 0.4 M KCl was then added dropwise and incubated with constant mixing at 4°C for 30 mins. The insoluble nuclear proteins were pelleted at 25,000 g for 2 mins.

### Immunofluorescence

myc-lacI-Lap2β expression was detected by immunofluorescence on cells fixed in 4% paraformaldehyde (pFa), with antibodies against the myc-tag (Upstate #06-549, 1∶100), LacI (clone 9A5, Upstate #05-503, 1∶100) and lap2 (Santa Cruz SC19784, 1∶100). FITC-conjugated anti-mouse and Texas Red anti-rabbit secondary antibodies were used at 1∶200 (Jackson ImmunoResearch Laboratories).

### 2D FISH

LacO plasmid and BAC/fosmid clones (Table S1) were labelled by nick translation with biotin-16-dUTP or digoxigenin-11-dUTP. 65-75 ng of labelled plasmid or 150 ng BAC were used per slide, together with 3 µg of human Cot1 DNA (GIBCO BRL) as competitor. Chromosome paints were from MP Biomedicals or Cambio. Cells were swollen in 75 mM KCl before fixation in 3∶1 methanol∶acetic acid. Hybridisation was as described previously [2]. After hybridisation, biotin-labelled probes were detected using fluorochrome-conjugated avidin (FITC or Texas Red) (Vector Laboratories) followed by biotinylated anti-avidin (Vector Laboratories) and a final layer of fluorochrome-conjugated avidin. Digoxigenin-labelled probes were detected with FITC anti-sheep (Vector). Slides were counterstained with 0.5 µg/ml DAPI.

### 3D Immuno-FISH

Cells grown on slides were permeabilised on ice for 5 mins in CSK buffer (100 mM NaCl, 300 mM sucrose, 3 mM MgCl2, 10 mM PIPES pH 6.8, 0.5% Triton X-100), fixed in 4% pFa at room temperature for 10 mins and then subject to freeze thaw in 20% glycerol/PBS, as previously described [1]. After thawing, the slides were washed in PBS and then incubated with 100 µg/ml RNaseA in 2×SSC for 1 hour at 37°C. Slides were denatured at 78°C in 70% deionised formamide/2×SSC for 3 mins followed by 50% formamide/2×SSC for 1 min. DNA probe preparation, denaturation, hybridisation and signal detection was as for 2D FISH. Slides were then incubated with antibody detecting lamin A (Santa Cruz SC6214, 1∶200), the epitope for which survives the FISH procedure.

### RNA FISH

RNase free PBS and H_2_O (0.1% DEPC-treated), and RNase Zap® were used to clean all containers used. Cells grown on slides were washed in RNAse-free PBS 3 times, and permeabilized on ice for 5 minutes in freshly made CSK Buffer containing 2 mM Vanadyl Ribonucleoside Complex) [38]. Cells were fixed in 4% pFa/RNase-free PBS for 10 minutes at room temperature then washed 3× in PBS. The slides were then washed in 70% ethanol for 5 minutes and stored in 70% ethanol at 4°C overnight. Slides were dehydrated through a 70%, 90%, 100% ethanol series for 3 minutes each and air-dried before hybridisation at 37°C overnight with 150 ng biotin-labelled pSV2-bsr. Slides were washed: 1× 3 mins 50% formamide/2×SSC at room temperature, 1× 3 mins 50%formamide/2×SSC at 37°C, 1× 3 mins 2×SSC at room temperature, 1× 3 mins 4×SSC/0.1 Tween 20 at room temperature. The RNA signal was then fixed by immersing the slides in 4% pFa for 15 mins then washing 3× in PBS.

### Image Capture and Analysis

Slides were examined on a Zeiss Axioplan II fluorescence microscope fitted with Plan-neofluar objectives, a 100 W Hg source (Carl Zeiss, Welwyn Garden City, UK) and Chroma #83000 triple band pass filter set (Chroma Technology Corp., Rockingham, VT) with the excitation filters installed in a motorised filter wheel (Prior Scientific Instruments, Cambridge, UK). Images were captured with a Coolsnap HQ CCD camera (Photometrics Ltd, Tucson, AZ). Image capture and analysis were performed using in-house scripts written for IPLab Spectrum (Scanalytics Corp, Fairfax, VA).

The radial positions of lacO sites, BAC clones and chromosome territories were determined as previously described [2] by radial analysis of 35–50 nuclei, using five shells of equal area eroded from the periphery (shell 1) through to the centre (shell 5) of the nucleus. The mean proportion (%) of hybridisation signal found in each shell was then normalised to the proportion of total DNA (DAPI stain) in each shell.

Stacks of images through the z-axis of cells subjected to 3D immuno-FISH were captured with a Zeiss Axioplan microscope fitted with a 100 watt mercury bulb, Ludl filter wheel, Chroma filter set #81000 and motorised stage attached to a cooled CCD Kodak KAF 1401e sensor camera (Princeton Instruments). A script was devised (P.Perry) using IPLAB v3.6 software (Scanlytics) to capture images at 0.5 µm intervals through the z-axis.

### Microarray Analysis

RNA was extracted using Trizol (Invitrogen) and quantified using a NanoDrop spectrophotometer (ND-1000). 40 ug of RNA were treated with DNase and cleaned up using Rneasy Columns (Qiagen). The RNA was then requantified and 20 ug of total treated RNA was used to create cDNA using the Superscript Direct cDNA module (Invitrogen) labelled with either Cy3 or Cy5 respectively. Differentially labelled RNAs from cell lines with and without the lacI-lap2β tether (tethered/untethered) were cohybridised to an oligonucleotide microarray representing ∼30,000 human genes (VUMC MACF human 30K oligo v60, GEO Accession: GPL5164) [39]. A minimum of four replicate hybridisations, incorporating both biological (separately isolated RNAs) and technical (dye-swap) replicates was carried out per cell line. Microarray slides were scanned under optimal scanning powers that produced a 1∶1 ratio of Cy3:Cy5 across ∼20% of the Microarray slide using the GenePix 4000B (Axon Instruments) connected to Genepix pro 4.1 scanning programme. Scanned images were Block Lowess normalised using Bluefuse v3.2 and the distributions of log2 (tethered/untethered) ratios were assessed from boxplots.

To combine the data from replicate experiments, the average log2 ratio tethered/untethered was then calculated for each oligo when the RNA from the tethered cell lines was Cy3 labelled and then for when this RNA was labelled with Cy5. A dye factor was generated by dividing the absolute (i.e. not log2) average Cy3: Cy5 ratios. The median and interquartile distances (75% quartile minus 25% quartiles) were calculated from the distribution of the log2 of this dye factor. Oligos were excluded from further analysis if they had a log2 dye factor >2× interquartile distance from the median. Outlying data points were then further excluded if the log2 tethered/untethered ratio was >2× interquartile distance from the median log2 of that oligo across all hybridisations. A mean log2 tethered/untethered ratio for each oligo was calculated from the remaining values [40]. Analysis along the genome was done using a moving average of 5 genes.

RNA from untethered cell lines was also used to create Cy3- and Cy5 labelled cDNAs that were hybridized (self-self hybridization) together onto the microarray slides (n = 4). This allowed experimental variation to be assessed and 99% confidence intervals were set from this at log2 = +/−0.5 for individual gene values and 0.35 for a moving average of 5 genes [33]. Individual p values of experimental data points were also set against the probability of finding these values in the self-self hybridization data.

### Real-Time PCR Analysis

The primers for real-time-PCR are detailed in Table S2. 5 ug RNA, prepared as described above, were used to create cDNA using the Superscript Direct cDNA module. All samples are diluted 1/100 before real time PCR analysis. Standard dilution curves were created for each primer set to ensure that they acted in a linear fashion. 10% decreases in cDNA samples (100, 90, 80 70, 60%) were used to test linearity of PCR primers Samples were analysed by real-time RT-PCR using a Quantitect® SYBR® Green detection kit (Qiagen) with a Peltier PTC-200 thermal cycler using an inbuilt Cromo4TM continuous fluorescence detector connected to Opticom 3.1 software interface. The real-time thermal cycler was programmed as follows: 15 min Hotstart; 44 PCR cycles (95°C for 15sec, 55°C for 30sec, 72°C for 20 sec). Melting curves were recorded from 60°C to 95°C and all PCR products revealed single bands, which were verified by 1.2% agarose gel electrophoresis. The pfaffl equation[41] was used to normalise the respective gene cycle threshold (*ct*) and linear phase PCR efficiency values from both control and lacI- lap2β tethered samples to those of β-actin. Each PCR reaction on a specific RNA sample was quantified three times, and also repeated on at least three independent cDNA samples. The microarray data sets used in this paper are available at NCBI:GEO under Accession number GSE10701.

### Addendum

A complementary study also demonstrates that transcription of genes can be repressed by tethering to the INM in mouse cells.

Reddy KL, Zullo JM, Singh H (2008) Transcriptional repression mediated by repositioning of genes to the nuclear lamina. doi:10.1038/nature06727.

## Supporting Information

Figure S1 Gene expression controls. (A) qRT-PCR analysis of endogenous genes on chromosome 4 (left) or chromosome11 (right), as well as the blasticidin gene that is in cis with the lacO sites, in the parental lacO-tagged cell lines, and then these cells now expressing a free nucleplasmic lacI. Graphs show the mean (+/−s.e.m) log2+/−free lacI ratios from independent analyses, normalised to β-actin. (B) qRT-PCR analysis of endogenous genes on chromosome 4 (left) or chromosome11 (right) analysed in the opposite cell lines (e.g. chromosome 4 genes in J21.C3 cells and chromosome 11 genes in B49.5 cells). Graphs show the mean (+/−s.e.m) log2tethered/untethered (i.e +/−lacI-lap2β) ratios from independent analyses, normalised to β-actin (white bars). Black bars indicate the log2 ratios obtained from the microarray analysis. Values along the x axis indicate estimated distance from lacO integration site in Mb (+ values are telomeric of the integration site, −ve values are centromeric).
(0.57 MB EPS)

Figure S2 Histone acetylation and nuclear organisation induced by TSA and sirtinol treatments. (A) Western blot with antibodies that detect either H4K5ac (top panel), or pan H4 (lower panel), on nuclear extracts from untreated (−) J21.C3 and J21.C3 lacI-lap2β cells, or cells treated with (+) 1 µM TSA or 10 µm sirtinol. (B) Histograms showing the mean proportion (%) of probe hybridisation signal, normalised to the proportion of DAPI stain (y axis), across the 5 concentric shells eroded from the periphery (shell 1) through to the centre (shell 5) of the nucleus (x axis), for a proximal BAC on the untagged chromosome (open bars) and on the tagged chromosome (black bars) and the lacO sites (red bars) in the lacI-lap2β expressing B49.5 and J21.C3 cell lines treated with DMSO (mock), TSA or sirtinol. n = 50 for each cell line.
(0.92 MB PPT)

Table S1 Genomic clones. Genomic position (start and end co = ordinates), in bp, of the genomic BAC and fosmid clones used for lacO integration site mapping in Figure 2B. Positions are from the March 2006 human reference sequence (NCBI Build 36.1) (http://genome.ucsc.edu/cgi-bin/hgGateway).
(0.04 MB DOC)

Table S2 PCR primers used for real time RT-PCR analysis of gene expression. Map position is from the March 2006 human reference sequence (NCBI Build 36.1) (http://genome.ucsc.edu/cgi-bin/hgGateway).
(0.07 MB DOC)
