# Supplementary material for: Recruitment to the Nuclear Periphery Can Alter Expression of Genes in Human Cells
Source: PLoS Genet. 2008 Mar 21;4(3):e1000039. doi: 10.1371/journal.pgen.1000039 (PMC2265557; doi:10.1371/journal.pgen.1000039)
Supplement: Figure S2 — Histone acetylation and nuclear organisation induced by TSA and sirtinol treatments. (A) Western blot with antibodies that detect either H4K5ac (top panel), or pan H4 (lower panel), on nuclear extracts from untreated (−) J21.C3 and J21.C3 lacI-lap2β cells, or cells treated with (+) 1 µM TSA or 10 µm sirtinol. (B) Histograms showing the mean proportion (%) of probe hybridisation signal, normalised to the proportion of DAPI stain (y axis), across the 5 concentric shells eroded from the periphery (shell 1) through to the centre (shell 5) of the nucleus (x axis), for a proximal BAC on the untagged chromosome (open bars) and on the tagged chromosome (black bars) and the lacO sites (red bars) in the lacI-lap2β expressing B49.5 and J21.C3 cell lines treated with DMSO (mock), TSA or sirtinol. n = 50 for each cell line. (0.92 MB PPT) [file pgen.1000039.s002.ppt]

## Slide 1
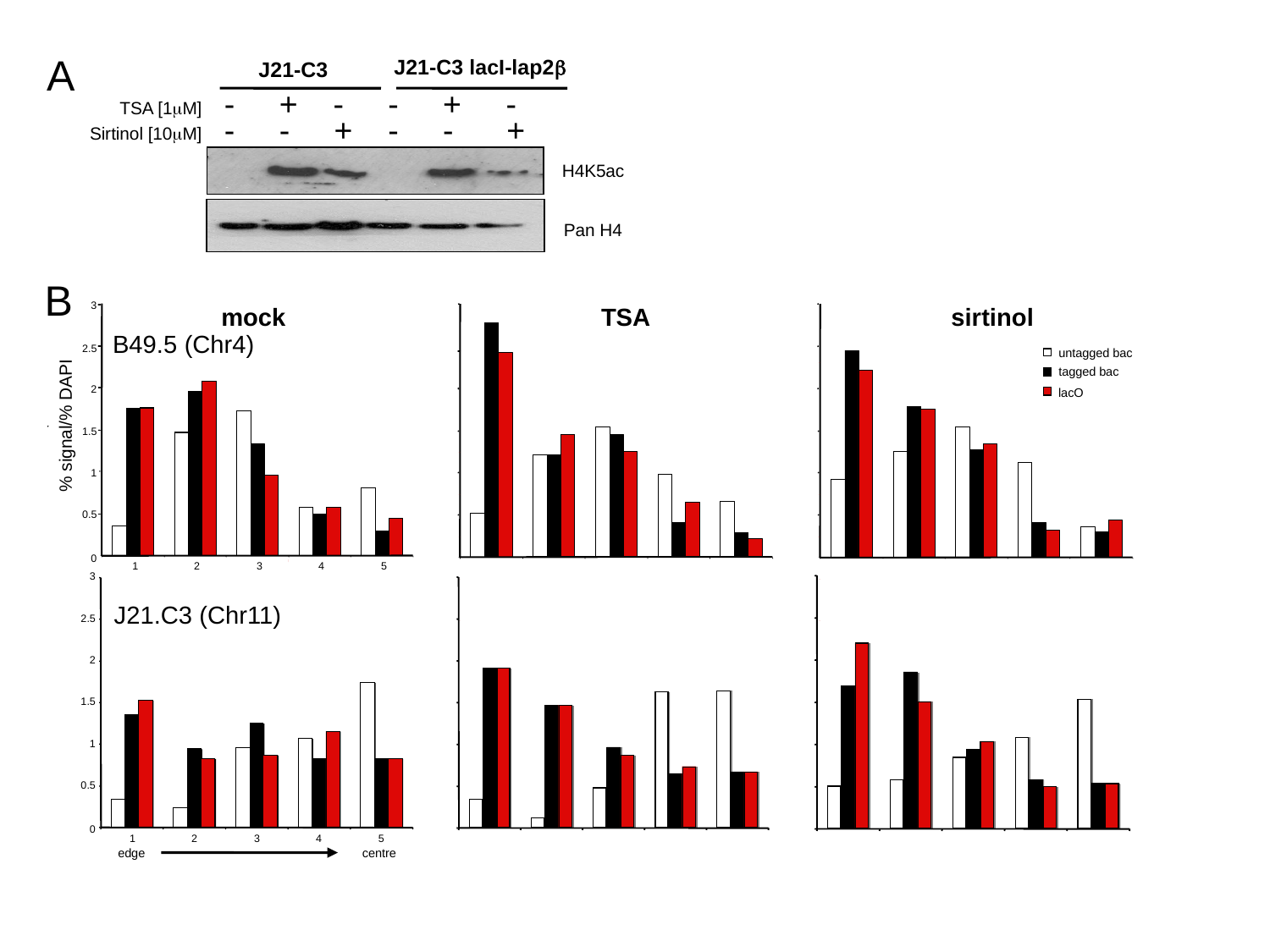

J21-C3
A
J21-C3 lacI-lap2β
- + - - + -
TSA [1μM]
Sirtinol [10μM]
- - + - - +
H4K5ac
Pan H4
B
mock
3
2.5
2
1.5
1
0.5
0
B49.5 (Chr4)
% signal/% DAPI
TSA
sirtinol
untagged bac
tagged bac
lacO
1
2
3
4
5
3
2.5
2
1.5
1
0.5
0
J21.C3 (Chr11)
1
2
3
4
5
edge
centre
