# Supplementary material for: Recruitment to the Nuclear Periphery Can Alter Expression of Genes in Human Cells
Source: PLoS Genet. 2008 Mar 21;4(3):e1000039. doi: 10.1371/journal.pgen.1000039 (PMC2265557; doi:10.1371/journal.pgen.1000039)
Supplement: Table S1 — Genomic clones. Genomic position (start and end co = ordinates), in bp, of the genomic BAC and fosmid clones used for lacO integration site mapping in Figure 2B. Positions are from the March 2006 human reference sequence (NCBI Build 36.1) (http://genome.ucsc.edu/cgi-bin/hgGateway). (0.04 MB DOC) [file pgen.1000039.s003.doc]

**Table S1.**

| **#chrom** | **chromStart** | **chromEnd** | **clone name** |
| --- | --- | --- | --- |
|  |  |  |  |
| chr4 | 125635479 | 125672866 | G248P82720G11 |
| chr4 | 127250446 | 127434541 | RP11-282B12 |
| chr4 | 128965677 | 129005986 | G248P80000A4 |
|  |  |  |  |
| chr11 | 65055439 | 65099774 | G248P800701F5 |
| chr11 | 65393334 | 65438020 | G248P88123B2 |
| chr11 | 65876916 | 66035381 | RP11-142G8 |
| chr11 | 66840191 | 66873614 | RP11-126P21 |
