# Supplementary material for: Recruitment to the Nuclear Periphery Can Alter Expression of Genes in Human Cells
Source: PLoS Genet. 2008 Mar 21;4(3):e1000039. doi: 10.1371/journal.pgen.1000039 (PMC2265557; doi:10.1371/journal.pgen.1000039)
Supplement: Table S2 — PCR primers used for real time RT-PCR analysis of gene expression. Map position is from the March 2006 human reference sequence (NCBI Build 36.1) (http://genome.ucsc.edu/cgi-bin/hgGateway). (0.07 MB DOC) [file pgen.1000039.s004.doc]

Table S2

|  | Gene name | **Gene Accession** | **Genome Position (bp)** | **Forward Primer 5'-3'** | **Reverse Primer 5'-3'** | **PCR product (b.p)** |
| --- | --- | --- | --- | --- | --- | --- |
|  | blasticidin | S81409 Brs |  | gtttgtgcagaagccatt | cacaaggacttaccactcg | 130 |
| **Chr 4** | DCK | NM_000788 | [72,096,866](../../../../E:%5Cpapersinprogress%5Ctethering%5Ccgi-bin%5ChgTracks%3Fdb=hg18&amp%3Bposition=chr4:72078256-72115477&amp%3Bhgsid=91671209&amp%3BknownGene=full) | aaacatatgcctgtctcagtcg | catccagtcatgccagtcttg | 197 |
|  | TIGD2 | NM_145715 | [90,254,033](../../../../E:%5Cpapersinprogress%5Ctethering%5Ccgi-bin%5ChgTracks%3Fdb=hg18&amp%3Bposition=chr4:90252991-90255075&amp%3Bhgsid=91671209&amp%3BknownGene=full) | aaacttttccctggcaat | cgagagtcgaaccactga | 146 |
|  | EGF | BC093731 | [111,103,183](../../../../E:%5Cpapersinprogress%5Ctethering%5Ccgi-bin%5ChgTracks%3Fdb=hg18&amp%3Bposition=chr4:111053499-111152868&amp%3Bhgsid=91671209&amp%3BknownGene=full) | tagcagttgatccagtagaaag | ctccatcataatcacaggagc | 219 |
|  | NUDT6 | NM_007083 | [124,048,229](../../../../E:%5Cpapersinprogress%5Ctethering%5Ccgi-bin%5ChgTracks%3Fdb=hg18&amp%3Bposition=chr4:124033249-124063209&amp%3Bhgsid=91671209&amp%3BknownGene=full) | gcagtacagaaatggagatcag | gcagtacagaaatggagatcag | 185 |
|  | AK057455 | AK057455 | [125,669,793](../../../../E:%5Cpapersinprogress%5Ctethering%5Ccgi-bin%5ChgTracks%3Fdb=hg18&amp%3Bposition=chr4:125640547-125699039&amp%3Bhgsid=91671209&amp%3BknownGene=full) | gggatgggtaagggataa | caggacctaagtcatttcca | 155 |
|  | FAT4 | NM_024582.3 | [126,544,695](../../../../E:%5Cpapersinprogress%5Ctethering%5Ccgi-bin%5ChgTracks%3Fdb=hg18&amp%3Bposition=chr4:126457017-126632373&amp%3Bhgsid=91671209&amp%3BknownGene=full) | acccagaacaattcttcaggtc | tgtaatcattaagacaaagcgctc | 186 |
|  | INTU | [NM_015693](http://www.ncbi.nlm.nih.gov/entrez/viewer.fcgi?db=nucleotide&amp;val=44888832) | [128,815,475](../../../../E:%5Cpapersinprogress%5Ctethering%5Ccgi-bin%5ChgTracks%3Fdb=hg18&amp%3Bposition=chr4:128773570-128857380&amp%3Bhgsid=91671209&amp%3BknownGene=full) | gagagaagtgttttcctcagc | ccacatatacacagtctggtc | 193 |
|  | AK090904 | AK090904 | [128,979,853](../../../../E:%5Cpapersinprogress%5Ctethering%5Ccgi-bin%5ChgTracks%3Fdb=hg18&amp%3Bposition=chr4:128978372-128981334&amp%3Bhgsid=91671209&amp%3BknownGene=full) | ggtttgctgaaaaacgttac | ttagggcatgaccgtaaat | 171 |
|  | NARG1 | NM_057175 | [140,486,755](../../../../E:%5Cpapersinprogress%5Ctethering%5Ccgi-bin%5ChgTracks%3Fdb=hg18&amp%3Bposition=chr4:140442126-140531385&amp%3Bhgsid=91671209&amp%3BknownGene=full) | ggttcgtagaggtttgagaaatg | cctcaagatctcgcatttgaatc | 196 |
|  | SMAD1 | NM_005900 | 146,661,088 | cgtttcctcactctcccaatag | gcgccatcatgtttgtgtcc | 196 |
|  | ETFDH | NM004453 | [159,812,904](../../../../E:%5CHomo_sapiens%5Ccontigview%3Fl=4:159812904-159849283) | ctccacttaacactcctgtaac | agcagctgcataaccagggta | 200 |
| **Chr 11** | Hras | NM_005343 | [523,896](../../../../E:%5Cpapersinprogress%5Ctethering%5Ccgi-bin%5ChgTracks%3Fdb=hg18&amp%3Bposition=chr11:522242-525550&amp%3Bhgsid=91671209&amp%3BknownGene=full) | tgccttctacacgttggt | aggagagcacacacttgc | 107 |
|  | BRSK2 | NM_003957 | [1,403,804](../../../../E:%5Cpapersinprogress%5Ctethering%5Ccgi-bin%5ChgTracks%3Fdb=hg18&amp%3Bposition=chr11:1367705-1439904&amp%3Bhgsid=91671209&amp%3BknownGene=full) | atagggggcaagaatgag | ctcctggttctcctcctc | 180 |
|  | POLR2G | NM_002696 | [62,288,174](../../../../E:%5Cpapersinprogress%5Ctethering%5Ccgi-bin%5ChgTracks%3Fdb=hg18&amp%3Bposition=chr11:62285591-62290757&amp%3Bhgsid=91671209&amp%3BknownGene=full) | aagtgtttccggtggatt | cttctgcttcaccgtgtt | 156 |
|  | BAD | NM_004322 | [63,801,309](../../../../E:%5Cpapersinprogress%5Ctethering%5Ccgi-bin%5ChgTracks%3Fdb=hg18&amp%3Bposition=chr11:63793878-63808740&amp%3Bhgsid=91671209&amp%3BknownGene=full) | acggaggacgacgaag | aaaggagtccacaaactcg | 138 |
|  | LTBP3 | NM_021070 | [65,072,428](../../../../E:%5Cpapersinprogress%5Ctethering%5Ccgi-bin%5ChgTracks%3Fdb=hg18&amp%3Bposition=chr11:65062850-65082006&amp%3Bhgsid=91671209&amp%3BknownGene=full) | aagcggacctgtctcaa | attcatgcaggggagag | 144 |
|  | EFEMP2 | NM_016938 | [65,393,670](../../../../E:%5Cpapersinprogress%5Ctethering%5Ccgi-bin%5ChgTracks%3Fdb=hg18&amp%3Bposition=chr11:65390488-65396852&amp%3Bhgsid=91671209&amp%3BknownGene=full) | tgaacgagtgtgacatgg | cagcggtactgacagagg | 162 |
|  | DIPA | NM_006848 | [65,415,066](../../../../E:%5Cpapersinprogress%5Ctethering%5Ccgi-bin%5ChgTracks%3Fdb=hg18&amp%3Bposition=chr11:65414451-65415682&amp%3Bhgsid=91671209&amp%3BknownGene=full) | tcatgcaggaggtgaatc | cttgggtccccaagag | 180 |
|  | DRAP1 | NM_006442 | [65,444,455](../../../../E:%5Cpapersinprogress%5Ctethering%5Ccgi-bin%5ChgTracks%3Fdb=hg18&amp%3Bposition=chr11:65443304-65445607&amp%3Bhgsid=91671209&amp%3BknownGene=full) | caagaagatcatgcagacg | atggtcttcgcgttcc | 144 |
|  | TNFRSF19L | NM_032871 | [72,775,610](../../../../E:%5Cpapersinprogress%5Ctethering%5Ccgi-bin%5ChgTracks%3Fdb=hg18&amp%3Bposition=chr11:72765053-72786167&amp%3Bhgsid=91671209&amp%3BknownGene=full) | gccaccgccagtct | cctcacaccgcaagttc | 190 |
|  | BC026292 | BC026292 | 81,372,800 | tcaggatggcagaagaga | cagggggttgtgtgataa | 123 |
|  | IL10RA | NM_001558 | [117,369,861](../../../../E:%5Cpapersinprogress%5Ctethering%5Ccgi-bin%5ChgTracks%3Fdb=hg18&amp%3Bposition=chr11:117362319-117377404&amp%3Bhgsid=91671209&amp%3BknownGene=full) | ccttggacctgtaccaca | tgaagccattgtggatct | 156 |
|  | AK095081 | AK095081 | [133,846,033](../../../../E:%5Cpapersinprogress%5Ctethering%5Ccgi-bin%5ChgTracks%3Fdb=hg18&amp%3Bposition=chr11:133811839-133880228&amp%3Bhgsid=91671209&amp%3BknownGene=full) | agccgttttcaggaaagt | aagaggaagcgttttggt | 101 |
